# On Boolean posets of numerical events

**DOI:** 10.1007/s43674-021-00004-w

**Published:** 2021-06-07

**Authors:** Dietmar Dorninger, Helmut Länger

**Affiliations:** 1 Faculty of Mathematics and Geoinformation, Institute of Discrete Mathematics and Geometry, TU Wien, Wiedner Hauptstraße 8-10, 1040 Vienna, Austria; 2 Faculty of Science, Department of Algebra and Geometry, Palacký University Olomouc, 17. listopadu 12, 771 46 Olomouc, Czech Republic

**Keywords:** Quantum effects, Numerical event, Quantum logic, Boolean poset, Set of states, 06C15, 03G12, 81P16

## Abstract

With many physical processes in which quantum mechanical phenomena can occur, it is essential to take into account a decision mechanism based on measurement data. This can be achieved by means of so-called numerical events, which are specified as follows: Let *S* be a set of states of a physical system and *p*(*s*) the probability of the occurrence of an event when the system is in state $s\in S$. A function $p:S\rightarrow [0,1]$ is called a numerical event or alternatively, an *S*-probability. If a set *P* of *S*-probabilities is ordered by the order of real functions, it becomes a poset which can be considered as a quantum logic. In case the logic *P* is a Boolean algebra, this will indicate that the underlying physical system is a classical one. The goal of this paper is to study sets of *S*-probabilities which are not far from being Boolean algebras by means of the addition and comparison of functions that occur in these sets. In particular, certain classes of so-called Boolean posets of *S*-probabilities are characterized and related to each other and descriptions based on sets of states are derived.

## Introduction

In axiomatic quantum mechanics, orthomodular partially ordered sets and generalizations of them are considered as “quantum logics” that determine the behaviour of a physical system. In particular, if the quantum logic is a Boolean algebra, then one will have reason to assume that one deals with a classical physical system. The elements of a quantum logic can also be interpreted as events, and a Boolean algebra then as the equivalent of a classical field of events as known from probability theory.

Having this in mind, we first recall the notion of a *numerical event* (cf. Beltrametti and Ma̧czyński (1991), Beltrametti and Ma̧czyński (1993) and Ma̧czyński and Traczyk (1973)).

Let *S* be a set of states of a physical system and *p*(*s*) the probability of the occurrence of an event, when the system is in state $s\in S$. The function *p* from *S* to [0, 1] is called a numerical event, or alternatively more precisely an *S-probability*. If ordered by the order $\le$ of functions and as the case may be endowed with some further properties, a set of *S*-probabilities becomes a partially ordered set (poset) that can be conceived as a quantum logic. In this paper, we study different kinds of such quantum logics, especially those that are not far away from being Boolean algebras. For this end, we provide the following notions.

Let *P* be a set of *S*-probabilities including the constant functions 0 and 1, partially ordered by the order of functions. We will call $p,q\in P$ *disjoint*, in symbols $p\wedge q=0$, if $x\le p,q$ for $x\in P$ implies $x=0$. Further, $p+q$ and $p-q$ shall denote the sum and difference of *p* and *q*, respectively, considered as real functions.

### Definition 1.1

A set *P* of *S*-probabilities is called *specific* if

1. $$0,1\in P$$,
2. if $$p\in P$$, then $$p':=1-p\in P$$,
3. if $p,q\in P$ and $p\wedge q=0$, then $p+q\in P$.

Condition (2) seems natural in respect to dealing with probabilities, and as for (3), this condition is motivated by classical fields of events (yet for the time being limited to considering the sum of disjoint events). Conceiving such a field of events as a Boolean ring *R* of subsets of some set $\Omega$, with $+$ the addition in *R*, one has $A{+}B=(A\cap B^c)\cup (A^c\cap B)$ for $A,B\in R$, where $\cup$ and $\cap$ stand for the set-theoretic join and meet (i.e. for union and intersection, respectively), $^c$ indicates complements in *R* and $\Omega$ has the role of the unity 1 of *R*. If *A* and *B* are disjoint, $A{+}B=A\cup B\in R$. Further, we observe that due to *R* having characteristic 2, i.e. $+$ will be the same as −, $1\varvec{-}A=\Omega {-}A=\Omega {+}A=A^c\in R$ in coincidence with condition (2).

Two *S*-probabilities *p* and *q* are called *orthogonal*, in symbols $p\perp q$, if $p\le q'$. From condition (3), it follows that $p\wedge q=0$ for $p,q\in P$ implies $p\perp q$. For orthoposets (which specific sets of *S*-probabilities in general are not), this property is known to be *Boolean* (cf., e.g. Tkadlec (1991); in connection with orthomodular posets see i.a. Ma̧czyński and Traczyk (1973) and Navara and Pták (1989)). We extend this definition to posets $(P,\le )$ with an *antitone involution*, i.e. a mapping $'$ from *P* to *P* such that $p\le q$ implies $p'\ge q'$ for $p,q\in P$ and $(p')'=p$ for $p\in P$.

### Definition 1.2

A poset *P* with an antitone involution is called *Boolean*, if $p\wedge q=0$ implies $p\perp q$ for $p,q\in P$.

According to this definition, specific sets of *S*-probabilities are Boolean posets.

Writing $p\vee q$ for the supremum of two elements *p*, *q* of a set *P* of *S*-probabilities and denoting their infimum by $p\wedge q$, we further point out

### Remark 1.3

Let *P* be a set of *S*-probabilities satisfying (1) and (2) and let $p,q\in P$. Then, De Morgan’s laws hold in *P* in the following sense: If $p\vee q$ exists in *P* then $p'\wedge q'$ exists in *P* and $(p\vee q)'=p'\wedge q'$, and if $p\wedge q$ exists in *P* then $p'\vee q'$ exists in *P* and $(p\wedge q)'=p'\vee q'$.

### Proof

Let $s\in P$. First assume $p\vee q$ to exist in *P*. Then, $(p\vee q)'\le p',q'$. If $s\le p',q'$, then $s'\ge p,q$ which means $s'\ge p\vee q$ from which we infer $s\le (p\vee q)'$. Hence, $p'\wedge q'$ exists in *P* and $p'\wedge q'=(p\vee q)'$. The second assertion follows by duality.

Finally, we recall the definitions of two structures of numerical events which we will later relate to specific sets of numerical events.

### Definition 1.4

A set *P* of *S*-probabilities is called a *generalized field of events* (in short GFE) (cf. Dorninger (2012)), if it satisfies (1), (2) and

(4) if $p,q\in P$ and $p\perp q$ then $p+q\in P$.

If a GFE satisfies

(5) if $p,q,r\in P$ and $p\perp q\perp r\perp p$ then $p+q+r\in P$,

then it is called an *algebra of* *S*-*probabilities* (cf. Beltrametti and Ma̧czyński (1991) and Beltrametti and Ma̧czyński (1993)).

Condition (4) is a special case of condition (5)—just assume *r* to be 0.

The goal of this paper is to characterize various classes of specific sets of *S*-probabilities, investigate their interrelations and closeness to Boolean algebras, and indicate when they will actually be Boolean algebras. Moreover, we will consider the question whether (small) sets of *S*-probabilities will belong to a Boolean subalgebra of a specific set of *S*-probabilities and we will characterize specific sets of *S*-probabilities by states.

## Specific sets of varying numerical events

### Definition 2.1

An *S*-probability *p* is called *varying*, if *p* is neither $\le 1/2$ nor $\ge 1/2$ unless $p=0$ or $p=1$.

The elements of an algebra of *S*-probabilities are varying (cf., e.g. Dorfer et al. (2010)), the elements of GFEs in general are not.

As for data won by experiments: That an *S*-probability is varying often comes up independently or can be achieved by adding further experimental data directed to this purpose.

Now, we will turn our attention to specific sets of *S*-probabilities that are varying. An *S*-probability is called *complementary* if $p\wedge p'=0$ (which by Remark 1.3 is equivalent to $p\vee p'=1$). A set *P* of *S*-probabilities with 0 and 1 will be called *complemented* if all of its elements are complementary. Further, we recall that a poset *P* with complementation $'$ which is an antitone involution is called an *orthoposet*.

### Proposition 2.2

A specific set *P* of varying *S*-probabilities has the following properties:

(i) *P* is complemented and hence an orthoposet,
(ii) if $p,q\in P$ and $p\perp q$, then $p\wedge q=0$,
(iii) *P* is a GFE.

### Proof

Let $$p,q,r\in P$$.

(i) If $r\ge p,p'$, then $r'\le p\le r$, from which we infer $r=1$ because of *r* being a varying *S*-probability. Therefore, $p\vee p'=1$ and hence, $p\wedge p'=0$.
(ii) If $p\perp q$ and $r\le p,q$, then because of $p\le q'$, we have $r\le q,q'$ and, since *P* being complemented, $r=0$ showing $p\wedge q=0$.
(iii) If $p\perp q$, then $p\wedge q=0$ according to (ii), and by condition (3), $p+q\in P$.

### Remark 2.3

Let *P* be a set of *S*-probabilities satisfying (1) and (2). Then, all elements of *P* are varying if and only if *P* is complemented.

### Proof

If all elements of *P* are varying, then *P* is complemented according to the proof of Proposition 2.2 (i). Conversely, assume *P* to be complemented. Let $p\in P$. If $p\le 1/2$, then $p\le p'$ and hence $p=p\wedge p'=0$. Dually, if $p\ge 1/2$, then $p'\le p$ and we get $p=p\vee p'=1$. This proves that every element of *P* is varying.

An orthoposet that allows a representation by a collection $\Delta$ of subsets of a set $\Omega$ such that

- $$\emptyset ,\Omega \in \Delta $$,
- if $$A\in \Delta $$ then $$\Omega \setminus A\in \Delta $$,
- if $A,B\in \Delta$ and $A\cap B=\emptyset$ then $A\cup B\in \Delta$,

is called a *concrete logic* (cf. Pták (2000)).

### Theorem 2.4

The specific sets of varying *S*-probabilities are exactly the complemented Boolean GFEs. They all are concrete logics.

### Proof

Let *P* be a set of *S*-probabilities and $p,q\in P$. First assume *P* to be a specific set of varying *S*-probabilities. By Proposition 2.2 (i) and (iii), *P* is a complemented GFE. Conversely, assume *P* to be a complemented Boolean GFE. Then, as mentioned in Remark 2.3, the elements of *P* are varying. Further, if $p\wedge q=0$, then $p\perp q$ and thus, $p+q\in P$ according to (4). By Proposition 2.2 (i), specific sets of varying *S*-probabilities are orthoposets and hence, Boolean GFEs are Boolean orthoposets. As mentioned in Tkadlec (1991), Boolean orthoposets are concrete logics due to a proof by Navara and Pták about Boolean orthomodular posets which does not make use of orthomodularity (cf. Navara and Pták (1989)).

Since any specific set of varying *S*-probabilities is a concrete logic, its elements can be represented by functions which have only the values 0 or 1. So, these *S*-probabilities must be varying from the outset. If *S* is finite, Theorem 2.4 leads to the conclusion that the specific sets of varying *S*-probabilities are Boolean algebras, since finite Boolean orthoposets are Boolean algebras. So to distinguish between a classical and a quantum mechanical behaviour by measurements in the form of numerical events, one would need data from *S*-probabilities for a continuous set *S* of states.

Next, we turn our attention towards the connection of specific sets of varying *S*-probabilities and algebras of *S*-probabilities.

### Lemma 2.5

The complemented Boolean GFEs are exactly the algebras of *S*-probabilities that are Boolean.

### Proof

According to Theorem 2.4, a complemented Boolean GFE is a concrete logic, and that such a GFE is an algebra of *S*-probabilities was already shown in Dorninger (2012). Conversely, every algebra of *S*-probabilities that is Boolean is also a Boolean GFE, and an arbitrary algebra of *S*-probabilities is complemented (because it is an orthoposet, first ascertained in Ma̧czyński and Traczyk (1973)).

In fact, algebras of *S*-probabilities are orthomodular posets with a full set of states, and vice versa (cf. Ma̧czyński and Traczyk (1973)). Further, an orthomodular poset is Boolean if and only if it is infimum faithful (cf. Godowski (1980)). To be *infimum faithful* means that $p\wedge q$ exists if and only if *p* and *q* *commute*, i.e. $p=(p\wedge q)\vee (p\wedge q')$. Since denoting an algebra of *S*-probabilities *P* as Boolean could be mixed up with *P* being a Boolean algebra, what in general is not the case, we rather prefer the notion infimum faithful. In the light of Theorem 2.4 and Lemma 2.5, we then obtain

### Theorem 2.6

The specific sets of varying *S*-probabilities are exactly the infimum faithful algebras of *S*-probabilities.

Returning to the motivation of the definition of specific sets of *S*-probabilities by Boolean rings, in line with Theorems 2.4 and 2.6, we can now remark:

### Remark 2.7

An infimum faithful algebra of *S*-probabilities which is a Boolean algebra can be conceived as a Boolean ring if one extends $+$ to arbitrary *S*-probabilities *p* and *q* by assuming within the pointwise addition of the functions *p* and *q* that $1+1=0$, and taking $p\cdot q:=p\wedge q$ for the ring’s multiplication.

## Further classes of specific sets of *S*-probabilities

Let *P* be a set of *S*-probabilities. We consider the following conditions:

(6) If $p,q\in P$ and $p\wedge q=0$ then $p+q=p\vee q\in P$,
(7) if $p,q,r\in P$, $p\perp q\perp r$ and $p\wedge r=0$ then $p+q+r\in P$,
(8) if $p,q,r\in P$, $p\perp q\perp r$ and $p\wedge r=0$ then $p+q+r\le 1$.

Condition (6) can be motivated by regarding a classical field of events as a Boolean ring *R* for which it is the case that $A\cap B=\emptyset$ for $A,B\in R$ implies $A{+}B=A\cup B$ (see Introduction). For short, we will denote specific sets of *S*-probabilities that satisfy condition (6) as $\vee$-*specific* (*join-specific*) sets of *S*-probabilities. If (1), (2) and (7) hold, *P* is called a *structured set of* *S*-*probabilities* (cf. Dorninger and Länger (2016)), and if (1), (2) and (8) are satisfied *P* is known as a *weakly structured set of* *S*-*probabilities* (cf. Dorninger and Länger (2016)).

Now, we define the following classes of sets of *S*-probabilities:

- $\mathcal C_1$: class of specific sets of *S*-probabilities,
- $\mathcal C_2$: class of $\vee$-specific sets of *S*-probabilities (satisfying (6)),
- $\mathcal C_3$: class of structured sets of *S*-probabilities (for which (7) is distinctive),
- $\mathcal C_4$: class of weakly structured sets of *S*-probabilities (characterized by (8)).

$\mathcal C_2$ is a subclass of $\mathcal C_1$, and this is also true for $\mathcal C_3$ as one can see by setting $q=0$ within (7).

### Lemma 3.1

We have $$\mathcal C_3\subseteq \mathcal C_2\subseteq \mathcal C_4$$.

### Proof

Let *P* be a set of *S*-probabilities and $p,q,r\in P$. First assume $P\in \mathcal C_3$. As already mentioned above, *P* is a specific set of *S*-probabilities. If $p\wedge q=0$, then $p+q=p\vee q$ because for $r\ge p,q$, we have $p\perp r'\perp q$ besides $p\wedge q=0$ from which we can conclude that $p+r'+q\in P$ showing that $p+q\le r$ and hence, $p+q=p\vee q$ which explains that $P\in \mathcal C_2$ and hence, $\mathcal C_3\subseteq \mathcal C_2$. Now, assume $P\in \mathcal C_2$, $p\perp q\perp r$ and $p\wedge r=0$. Since $p\le q'$ and also $r\le q'$, we obtain that $p+r=p\vee r\le q'$ from which we infer $p+q+r\le 1$. Therefore, $P\in \mathcal C_4$ and hence, $\mathcal C_2\subseteq \mathcal C_4$.

### Lemma 3.2

We have $$\mathcal C_2=\mathcal C_1\cap \mathcal C_4$$.

### Proof

Let *P* be a specific set of *S*-probabilities which is also a weakly structured set of *S*-probabilities and assume $p,q,r\in P$ such that $p\wedge q=0$ and $r\ge p,q$. Then, $p\perp r'\perp q$ and hence, $p+r'+q\le 1$, i.e. $p+q\le r$ which shows $p+q=p\vee q$. Since according to Lemma 3.1$\mathcal C_2\subseteq \mathcal C_4$, we are done.

### Theorem 3.3

The class of structured sets of *S*-probabilities is a proper subclass of the class of $\vee$-specific sets of *S*-probabilities which on its part is a proper subclass of the class of weakly structured sets of *S*-probabilities unless one assumes that only specific sets of *S*-probabilities are taken into account.

### Proof

As for the inclusions to be proper, in agreement with Lemma 3.1, it suffices to consider the following two examples:

First, assume $|S|=2$ and define

$$\begin{aligned} P:= & {} \{(0,0),(1/4,1/4),(1/4,3/4),(3/4,1/4),\\&(3/4,3/4),(1,1)\}. \end{aligned}$$

Then, $P\in \mathcal C_2$, but $P\notin \mathcal C_3$ since

$$\begin{aligned}&(0,0)\perp (1/4,1/4)\perp (1/4,3/4)\text { and }\\&\quad (0,0)\wedge (1/4,3/4)=(0,0), \end{aligned}$$

but

$$\begin{aligned} (0,0)+(1/4,1/4)+(1/4,3/4)=(1/2,1)\notin P. \end{aligned}$$

Second example: Again we assume $|S|=2$ and this time define

$$\begin{aligned} P:=\{(0,0),(0,1/2),(1/2,0),(1/2,1),(1,1/2),(1,1)\}. \end{aligned}$$

Then, $P\in \mathcal C_4$, but $P\notin \mathcal C_2$ since

$$\begin{aligned}&(0,1/2)\wedge (1/2,0)=(0,0),\text { but }\\&\quad (0,1/2)+(1/2,0)=(1/2,1/2)\notin P. \end{aligned}$$

That the $\vee$-specific sets of *S*-probabilities are exactly the elements of $\mathcal C_1\cap \mathcal C_4$ is confirmed by Lemma 3.2.

Next we will discuss the question how far $\vee$-specific sets of *S*-probabilities are away from being Boolean algebras. A first reference to this can be a subclass of $\mathcal C_3$.

### Theorem 3.4

(cf. Dorninger and Länger (2016)) The complemented members of the class $\mathcal C_3$ of structured sets of *S*-probabilities are exactly the infimum faithful algebras of *S*-probabilities.

Though $\mathcal C_3$ is a proper subclass of $\mathcal C_2$, more incisive properties have to be taken into account to distinguish $\mathcal C_2$ from Boolean algebras: e.g. if a complemented structured set of *S*-probabilities *P* is finite, it is a Boolean algebra, because, as already mentioned, finite Boolean orthoposets are Boolean algebras. Further, *P* is a Boolean algebra if it is orthocomplete (cf. Tkadlec (1994)). (To be orthocomplete means that the supremum of any set of pairwise orthogonal elements of *P* has to belong to *P*.) Moreover, if *P* is a lattice (i.e. $p\vee q$ and $p\wedge q$ exist for all $p,q\in P$), then we also have a Boolean algebra (cf. Tkadlec (1991)). That *P* is lattice-ordered can be characterized by a simple criterion: According to Theorem 2.4, *P* is a concrete logic, and as shown in Dorninger and Länger (2016), a structured set of *S*-probabilities *P* which is a concrete logic is a lattice if and only if for all $p,q\in P$ $\max (p,q)\in P$ (the maximum of the functions considered pointwise).

There are many papers in which (arbitrary) classes of algebras of *S*-probabilities are characterized to be Boolean algebras by specifying some structural properties—for an overview of these papers see Dorninger (2020)—and there are numerous results on Boolean orthoposets and concrete logics which can all be applied to fathom the distance between specific sets of *S*-probabilities and Boolean algebras (cf. i.a. Klukowski (1975), Navara and Pták (1989), Pták (2000), Tkadlec (1991), Tkadlec (1994) and Tkadlec (1997)).

Sometimes it is not of interest if a whole algebra of *S*-probabilities *P* is a Boolean algebra but if a (usually small) subset of *P* belongs to a Boolean subalgebra of *P*. If this were the case, this would indicate that one locally deals with a classical physical system. To answer this question, the existence of some further *S*-probabilities in *P* will have to be asked for, but the knowledge of *P* in detail will not be important.

So let us assume that a subset $\{p_1,\ldots ,p_n\}$ of a known or hypothetically assumed infimum faithful algebra of *S*-probabilities *P* is given. If $p_1,\ldots ,p_n$ are pairwise orthogonal, then there does exist a Boolean subalgebra of *P* wherein $p_1,\ldots ,p_n$ are contained, as it is well known for every subset of mutually orthogonal elements of an orthomodular poset (cf. Dorninger et al. (2020)), and as proved in Ma̧czyński and Traczyk (1973), every algebra of *S*-probabilities is orthomodular. So, let us suppose that $\{p_1,\ldots ,p_n\}$ is an arbitrary subset of *P*.

Having in mind that the elements of *P* can only assume the values 0 and 1 (cf. Theorems 2.4 and 2.6) and defining $p\cdot q$ for $p,q\in P$ by $(p\cdot q)(s)=p(s)\cdot q(s)$ for $s\in S$, one obtains that if $p\cdot q$ exists in *P*, then $p\cdot q=p\wedge q$. It is obvious then that $p^k=p$ for $k=1,2,3,\ldots$ and that the multiplication is associative.

In Section 2, we have defined what it means that *p* and *q* commute. We will express this fact by writing $p\mathrel {\text {C}}q$ and point out that for orthomodular posets, $p\mathrel {\text {C}}q$ is equivalent to $q\mathrel {\text {C}}p$. Further, we agree to write $\bigwedge B$ for the infimum of the elements of a finite subset *B* of *P*. Now, we can prove the following

### Theorem 3.5

The set $\{p_1,\ldots ,p_n\}$ is contained in a Boolean subalgebra of an infimum faithful algebra of *S*-probabilities *P* if and only if $p_{i_1}\cdot \cdots \cdot p_{i_n}\in P$ for all $i_1,\ldots ,i_n\in \{1,\ldots ,n\}$.

### Proof

Assume $n=2$. Then according to Theorem 3.4, in Dorninger et al. (2020), $\{p_1,p_2\}$ is contained in a Boolean subalgebra of *P* if and only if $p_1\barwedge p_2(:=\min (p_1,p_2))\in P$ which in our notion means that $p_1\cdot p_2\in P$. In this theorem, it is also stated that $p_1\barwedge p_2(=p_1\cdot p_2)\in P$ is equivalent to $p_1\mathrel {\text {C}}p_2$.

Next we make use of Corollary 2.3 in Dorninger and Länger (2014) which says: Let *A* be a subset of an orthomodular poset *P* with $n>1$ elements. Then, *A* is contained in an Boolean subalgebra of *P* if and only if $(\bigwedge B)\mathrel {\text {C}}(\bigwedge D)$ for every $k\in \{1,\ldots ,n-1\}$ and every *k*-element subsets *B* and *D* of *A*.

Now, we assume *P* to be our structured set of *S*-probabilities and $A=\{p_1,\ldots ,p_n\}\subseteq P$. Then, $\bigwedge B$ and $\bigwedge D$ are the products $p_B$ and $p_D$ of the elements of *B* and *D*, respectively. If $p_{i_1}\cdot \cdots \cdot p_{i_n}\in P$ for all $i_1,\ldots ,i_n\in \{1,\ldots ,n\}$, then $p_B\mathrel {\text {C}}p_D$ for every subsets *B* and *D* of *A* with $k\le n-1$ elements since $p_B\cdot p_D$ is an element of *P* and, as mentioned above, $p_B\mathrel {\text {C}}p_D$ is equivalent to $p_B\cdot p_D\in P$. Thus, we can conclude that the elements of *A* are contained in a Boolean subalgebra of *P*. The converse is obvious.

Besides the possibility to describe sets of *S*-probabilities by structural properties, one can also try to characterize them by states, as was done by M. J. Ma̧czyński and T. Traczyk, who characterized algebras of *S*-probabilities as the orthomodular posets which have a full set of states (cf. Ma̧czyński and Traczyk (1973)).

## Algebraic representations of specific sets of *S*-probabilities

We begin by extending the commonly known notion of a state to the class of bounded posets *P* with an antitone involution.

### Definition 4.1

A *specific state* on a bounded poset $P=(P,\le ,{}',0,1)$ with an antitone involution is a mapping *s* from *P* to [0, 1] satisfying the following conditions for all $p,q\in P$:

1. $$s(0)=0$$ and $$s(1)=1$$,
2. $$s(p')=1-s(p)$$,
3. if $$p\le q$$, then $$s(p)\le s(q)$$,
4. if $p\wedge q=0$, then there exists some $r\in P$ with $r\ge p,q$ and $s(r)=s(p)+s(q)$.

If for $p,q\in P$ with $p\wedge q=0$ the element $p\vee q$ exists in *P*, then a specific state on *P* satisfying

(S5) if $p,q\in P$ and $p\wedge q=0$ then $s(p\vee q)=s(p)+s(q)$,

is called a *pseudostate* on *P* (cf. Dorninger and Länger (2016)).

A *set* *T* *of specific states* on *P* is called *full* if for $p,q\in P$, $s(p)\le s(q)$ for all $s\in T$ implies $p\le q$, and a *set* *T* *of specific states* on *P* is called *uniform* if for disjoint $p,q\in P$ condition (S4) is satisfied for all $s\in T$ with the very same *r*. (With pseudostates, one can take $r=p\vee q$.)

### Theorem 4.2

Up to isomorphism, the specific sets of *S*-probabilities are exactly the bounded posets with an antitone involution having a full and uniform set of specific states.

### Proof

Let $P=(P,\le ,{}',0,1)\in \mathcal C_1$ with $P\subseteq [0,1]^S$, $a\in S$ and $p,q\in P$. Then, clearly, *P* is a bounded poset with an antitone involution. We define $s_x(r):=r(x)$ for all $x\in S$ and $r\in P$. Then, we have

1. $$s_a(0)=0(a)=0$$ and $$s_a(1)=1(a)=1$$,
2. $$s_a(p')=p'(a)=1-p(a)=1-s_a(p)$$,
3. if $$p\le q$$ then $$s_a(p)=p(a)\le q(a)=s_a(q)$$,
4. if $p\wedge q=0$ then $p+q\in P$, $p+q\ge p,q$ and $s_a(p+q)=(p+q)(a)=p(a)+q(a)=s_a(p)+s_a(q)$.

Further, if $s_x(p)\le s_x(q)$ for all $x\in S$, then $p\le q$. Hence, $\{s_x\mid x\in S\}$ is a full and uniform set of specific states on *P*.

Conversely, let $P=(P,\le ,{}',0,1)$ be a bounded poset with an antitone involution which has a full and uniform set *S* of specific states and let $p,q,r\in P$ and $s\in S$. We define $(f(u))(t):=t(u)$ for all $u\in P$ and $t\in S$. Then, the following assertions are equivalent: $f(p)\le f(q)$, $(f(p))(t)\le (f(q))(t)$ for all $t\in S$, $t(p)\le t(q)$ for all $t\in S$, $p\le q$. Therefore $f(p)=f(q)$ if and only if $p=q$. Next we will prove $f(P)\in \mathcal C_1$:

1. $(f(0))(s)=s(0)=0$ and $(f(1))(s)=s(1)=1$; thus, $0=f(0)\in f(P)$ and $1=f(1)\in f(P)$.
2. $$(f(p'))(s)=s(p')=1-s(p)=1-(f(p))(s)=(f(p))'(s)$$; therefore, $$(f(p))'=f(p')\in f(P)$$.
3. Assume $f(p)\wedge f(q)=f(0)$. If $r\le p,q$, then $f(r)\le f(p),f(q)$, from which we infer $f(r)=f(0)$, i.e. $r=0$, showing $p\wedge q=0$. Accordingly, there exists some $u\in P$ (which is independent of *s*) with $s(u)=s(p)+s(q)$. Now,
  $$\begin{aligned}&(f(p)+f(q))(s)=(f(p))(s)+(f(q))(s)\\&\quad =s(p)+s(q)=s(u)=(f(u))(s), \end{aligned}$$
  i.e. $$f(p)+f(q)=f(u)\in f(P)$$.

From this, we can conclude that $f( P)=(f(P),\le ,{}',0,1)\in \mathcal C_1$ and that *f* is an isomorphism from *P* onto *f*(*P*). Hence, *P* is isomorphic to a member of $\mathcal C_1$.

As shown in Dorninger and Länger (2016) up to isomorphism, the weakly structured sets of *S*-probabilities are exactly the bounded posets with an antitone involution in which the join of two disjoint elements exists and which have a full set of pseudostates, which in the light of Lemma 3.2 then reads

### Theorem 4.3

Up to isomorphism, the $\vee$-specific sets of *S*-probabilities are exactly the bounded posets with an antitone involution in which the sum of two disjoint elements equals their join and which have a full set of pseudostates.

Theorems 4.2 and 4.3 are analogues to the theorem mentioned above that up to isomorphism, the algebras of *S*-probabilities are exactly the orthomodular posets having a full set of states.

## Conclusions

To find out whether quantum mechanical effects can occur in a physical or technical system, various classes of sets of numerical events were studied, i.e. data-sets obtained by surveying the system which then form or contribute to the underlying quantum logic. In particular, sets of numerical events, called specific sets, were analyzed that are located at the threshold between classical and quantum behavior, a situation that can be characterized by the fact that in case of a classical physical system the underlying logic is a Boolean algebra. The interrelations of specific sets of numerical events and their closeness to Boolean algebras were ascertained and the question was answered when an unstructured set of numerical events can be a part of a Boolean algebra. Moreover, specific sets of numerical events were characterized by the states they admit.
